# Clinical characteristics of young-age onset gastric cancer in Korea

**DOI:** 10.1186/s12876-016-0528-y

**Published:** 2016-09-06

**Authors:** Jieun Lee, Myung Ah Lee, In-Ho Kim, Sang-Young Roh

**Affiliations:** 1 Division of Medical Oncology, Department of Internal Medicine, Seoul St. Mary’s Hospital, College of Medicine, The Catholic University of Korea, 222 Banpo-daero, Seocho-gu, Seoul, 06591 South Korea; 2 Cancer Research Institute, College of Medicine, The Catholic University of Korea, Seoul, South Korea

**Keywords:** Young age cancer, Gastric cancer

## Abstract

**Background:**

Gastric cancer is the fourth most common cancer worldwide and more frequently detected in Asian countries including Korea and Japan. The incidence of young-age gastric cancer (GC) is increasing worldwide, but clinical behavior of young-age GC patients is not well established. We retrospectively analyzed the clinical features and outcomes of GC diagnosed at young-age population.

**Methods:**

Between Jan. 2009 to Jan. 2015, 163 patients diagnosed as early, advanced, recurrent, or metastatic GC at ages between 22 ~ 39 years were analyzed. Based on medical records, authors analyzed the clinicopathologic characteristics and survival outcomes including overall survival (OS), disease free survival (DFS), and progression free survival (PFS).

**Results:**

One-hundred and four patients (82.8 %) were diagnosed as GC at their thirties; especially 81 patients (31.2 %) patients were diagnosed over 35 years of age. The ratio of early GC and advanced GC were relatively similar (47.2 % vs. 52.8 %, respectively). Among stage II and III patients, 45 patients received 5-FU based adjuvant chemotherapy and recurrence rate was 48.9 %. Among patients diagnosed as recurrent or metastatic GC, recurrent GC patients showed relatively superior PFS and OS after cancer recurrence, compared to metastatic GC patients, but without statistical significance. Among metastatic GC patients, patients receiving palliative debulking surgery for ovary metastases showed superior PFS compared to patients who only received palliative systemic chemotherapy (*P* = 0.021, PFS 7.7 vs. 3.37 months, respectively).

**Conclusions:**

Young age GC were commonly diagnosed at their thirties, without sexual predominance. The incidence of advanced GC in young age patients were higher compared to general patient population. Among recurrent GC patients, palliative debulking surgery might have role for superior survival outcomes. Considering relatively higher incidence for advanced GC, active surveillance for gastric cancer is warranted.

## Background

Gastric cancer (GC) is the fourth most common in cancer prevalence, and second leading cause of cancer-related death worldwide [1]. Although the incidence of GC is decreasing in Western, the incidence of GC remains high in Asia. In Korea, GC is the most common cancer in incidence among male population and fourth most common in female population [2]. Mean age of GC in Korea and Japan is over 50 years of age [3], but there are some proportions of patients who are diagnosed as GC in young age. The definition of young age GC remains controversial, but mainly other literatures defined young age below 40 years of age [4, 5].

The incidence and clinicopathologic characteristics of young age GC is relatively not well defined. The incidence of young age GC ranges from 2–15 % [6–9], with controversial results about prognosis. Previous studies have reported poor prognosis of young age GC patients compared to general population [10, 11], but other studies report equivalent or better prognosis between young and elderly GC patients [5, 12]. The clinical characteristics of young age GC is analyzed based on a heterogeneous patient population with regard to races and different definitions of young age population, resulting to various results.

In our analysis, authors have analyzed the clinical characteristics and survival outcomes of GC diagnosed in our center. Furthermore, authors have also analyzed the clinical features and survival outcomes of patients with recurrent or metastatic GC diagnosed at a young age.

## Methods

### Patients

From January 2009 to January 2015, the medical records of patients diagnosed and treated as gastric cancer in Seoul St. Mary’s hospital were retrospectively reviewed. In 5 years of following, 4,333 patients were diagnosed as gastric cancer. Among these patients, 163 patients were diagnosed as young age gastric cancer, age ranging in 22 to 39 years. The other eligible criteria were as follows: (1) pathologically confirmed as adenocarcinoma by endoscopic biopsy or surgical specimen; (2) patients who regularly followed up in Seoul St. Mary’s Hospital. We analyzed the clinical characteristics, laboratory findings, surgical treatment option, systemic chemotherapy regimens and survival outcomes through the medical records. The Gross and microscopic pathologic findings were reviewed based on operation records and pathology reports. This study was approved by the Institutional Review Board (IRB) of Seoul St. Mary’s Hospital, Catholic University of Korea (KC15RISI0675).

### Treatments

Patients who were diagnosed as early gastric cancer underwent endoscopic mucosal resection or primary surgical resection. Patients with stage II or III gastric cancer received primary surgical resection followed up by 5-FU based adjuvant systemic chemotherapy. Patients with stage IV gastric cancer were treated with oxaliplatin based systemic chemotherapy (FOLFOX, XELOX) as first line treatment. Among patients who showed progression after first line chemotherapy with good performance status, irinotecan or docetaxel based systemic chemotherapy were given. Monthly physical examination with laboratory evaluation were performed in early or locally advanced gastric cancer who received curative surgical resection with or without adjuvant chemotherapy. When cancer recurrence was detected, patients were treated as stage IV gastric cancer. Patient who showed cancer recurrence as Krukenberg tumor, resectability of metastatic ovary lesions were discussed with gynecologic surgeons in multidisciplinary meetings. Patients who underwent metastasectomy, systemic chemotherapy were administered as stage IV gastric cancer. Response evaluation was performed by CT scans every 2 to 3 months according to Response Evaluation Criteria In Solid Tumors (RECIST) criteria, ver. 1.0. Systemic chemotherapy was administered until unaccepted toxicity, disease progression or patients’ refusal.

### Statistical analysis

Overall survival (OS) was calculated from the date of initial diagnosis of gastric cancer to the death of patient or patient’s last follow-up date. For patients who showed cancer recurrence, overall survival (OS) after recurrence was determined from the date of recurrence confirmed by radiologic studies to patient’s death, or last follow-up date. Disease free survival (DFS) was calculated from the date of surgical resection to the date of disease recurrence, confirmed by CT scans. First progression free survival (first-line PFS) was measured from the first administration date of first line systemic chemotherapy to the date of disease progression, confirmed by CT scans. OS, OS after cancer recurrence, DFS, and first-line PFS were analyzed using log-rank test and Kaplan-Meier method. All statistical analyses were performed with SPSS (ver. 18.0).

## Results

### Patient’s characteristics

During 5 years of follow-up in Seoul St. Mary’s hospital, 4,333 patients were diagnosed as early or advanced gastric cancer and 163 patients (3.76 %) were diagnosed before age of 40. Baseline characteristics of patient population are described in Table 1. The median age was 35 years, with even male to female ratio. Most patients (49.7 %) were diagnosed over age of 36 years, but 29 patients (17.8 %) were diagnosed before age of 30. There was no any other primary cancer diagnosed in same patient in our analysis. One hundred and eighteen patients (72.4 %) had no familial cancer history, with 19 patients (11.7 %) with familial history of gastric cancer. The predominant primary cancer occurring site was gastric body (66.3 %). The proportion of early gastric cancer (EGCa) and advanced gastric cancer (AGCa) were relatively similar, with 47.2 % for EGCa and 52.8 % for AGCa. In total patient population, 90 patients (55.2 %) showed signet ring cell carcinoma. Irrespective of cancer stage, signet ring cell (SRC) carcinoma showed about 50 % of predominance. There was female predominance (46 patients, 58 %) in patients who were diagnosed as SRC.

**Table 1:** Characteristics of patient population

	No. (%)
No. of patients	163
Sex	
Male : Female	80 (49.1) : 83 (50.9)
Age (years)	median 35 (range 22 ~ 39)
22 ~ 30	29 (17.8)
31 ~ 35	53 (31.5)
36 ~ 39	81 (49.7)
Smoking	
Never smoker	111 (68.1)
Ex-smoker	7 (4.3)
Current smoker	45 (27.6)
Body mass index	median 21.89 (range 15.25 ~ 29.76)
< 18.5	16 (9.8)
≥ 23.0	56 (34.4)
Blood type	
A/B/O/AB	79 (48.5)/27 (16.6)/36 (22.1)/21 (12.8)
Familial history (including second degree)	
Gastric cancer	19 (11.7)
Others	22 (8.4)
None	118 (72.4)
unknown	4 (7.5)
Location	
Antrum	39 (23.9)
Body	108 (66.3)
Upper body & Cardia	7 (4.3)
Diffuse	1 (0.6)
Not assessed	8 (4.9)
^*^Early gastric cancer	77 (47.2)
^**^Advanced gastric cancer	86 (52.8)
Initial tumor stage (AJCC 7th edition)	
I	80 (49.1)
II	26 (16)
III	24 (14.7)
IV	33 (20.2)
^***^CEA level	assessed = 133 patients
≤ 3 ng/mL	121 (91)
> 3 ng/mL	12 (9) median 4.935 (range 3.62-477.5)
H.pylori	assessed = 57 patients
positive	37 (65)
negative	20 (35)

^*^ Early gastric cancer: cancer confined to mucosa or submucosa, irrespective of lymph nodes

^**^ Advanced gastric cancer: cancer invading muscularis propria, irrespective of lymph nodes

^***^ CEA: Carcinoembryonic antigen

### Treatment and clinical outcomes

Patients who were diagnosed as stage II or stage III gastric cancer were recommended for adjuvant chemotherapy. Among 50 patients, 45 patients (90 %) received adjuvant chemotherapy (Table 2). Twenty-five patients (55.6 %) received S-1 as adjuvant treatment. Four patients (8.9 %) were treated with capecitabine-oxaliplatin (XELOX) regimen, and 13 patients (28.9 %) received platinum based doublet regimen as adjuvant treatment.

**Table 2:** Characteritsics of patients who received adjuvant chemotherapy

	No. (%)
No. of patients	45
Pathologic staging	
II	23 (51.1)
III	22 (48.9)
Regimen	
S1	25 (55.6)
XELOX	4 (8.9)
5-FU based doublet	13 (28.9)
unknown	1 (6.6)
Recurrence	22 (48.9)
II	7 (30.4)
III	15 (68.2)

The recurrence rate among stage II or III gastric cancer was 48.9 %. Seven patients (26.9 %) showed recurrence among stage II gastric cancer. Among stage III gastric cancer, 15 patients (68.2 %) showed disease recurrence during follow-up. Median DFS was 24.29 months (range 7.13 months ~ 13.7 years) among stage II and III GC, and median DFS of stage III GC was 15.13 months (range 7.13 months ~ 13.7 years). Ten patients (43.5 %) who showed disease recurrence were diagnosed as signet ring cell carcinoma by pathologist.

### Characteristics and survival outcomes in recurrent or stage IV gastric cancer

Thirty-three patients were initially diagnosed as stage IV gastric cancer, and 23 patients showed recurrent gastric cancer (Table 3). The median age was 35 years (range 22 ~ 39 years). Twelve patients were diagnosed as recurrent or stage IV gastric cancer at their twenties.

**Table 3:** Characteristics of patients with recurrent or metastatic GC

	No. (%)
No. of patients	56
Sex	
Male	24 (42.9)
Female	32 (57.1)
Age (years)	
Median	35
Range	22 ~ 39
Recurrence	23 (41.1)
Initial stage IV	33 (58.9)
Pathology	
Adenocarcinoma	27 (48.2)
Signet ring cell carcinoma	27 (48.2)
Not assessed	2 (3.6)
Recurrence or metastatic site	
Bone	13 (23.2)
Lymphangitic lung metastasis	6 (10.7)
Ovary	14 (25)
Carcinomatosis peritonei	31 (55.4)
Liver	2 (3.6)
Lymph node	20 (35.7)
Pericardium	2 (3.6)
Leptomeninges	3 (5.4)
Bone marrow	3 (5.4)
Anastomosis site	5 (8.9)
Treatment after diagnosis or recurrence	
Chemotherapy (5-FU based)	21 (37.5)
Intrathecal chemotherapy	1 (1.8)
Debulking surgery	3 (5.4)
Palliative care	2 (3.6)
Follow-up loss	2 (3.6)

Fifty-one patients received 5-FU based systemic chemotherapy after detection of stage IV or recurrent gastric cancer. Oxaliplatin and 5-FU combination chemotherapy was most commonly administered as first line treatment, followed up by irinotecan and 5-FU combination chemotherapy as 2nd line treatment. Forty-nine patients (96.1 %) received first line chemotherapy, and 39 patients (76.4 %) received second line chemotherapy. Survival outcomes are described at Table 4. Recurrent gastric cancer patients showed relatively superior PFS during first-line chemotherapy (first-line PFS) and OS after cancer recurrence compared to first-line PFS and OS of stage IV gastric cancer group, but without statistical significance.

**Table 4:** Survival outcomes of recurrent & metastatic stage IV gastric cancer

Recurrent gastric cancer	
Overall survival after recurrence (range)	13.5 months (1.06~97.1 months)
1st progression free survival (range)	5.57 months (0.7~62.5 months)
Metastatic gastric cancer	
Overall survival (range)	10.5 months (1.7 mo.~8.5 years)
1st progression free survival (range)	3.8 months (0.47~19.8 months)

**Fig. 1:**
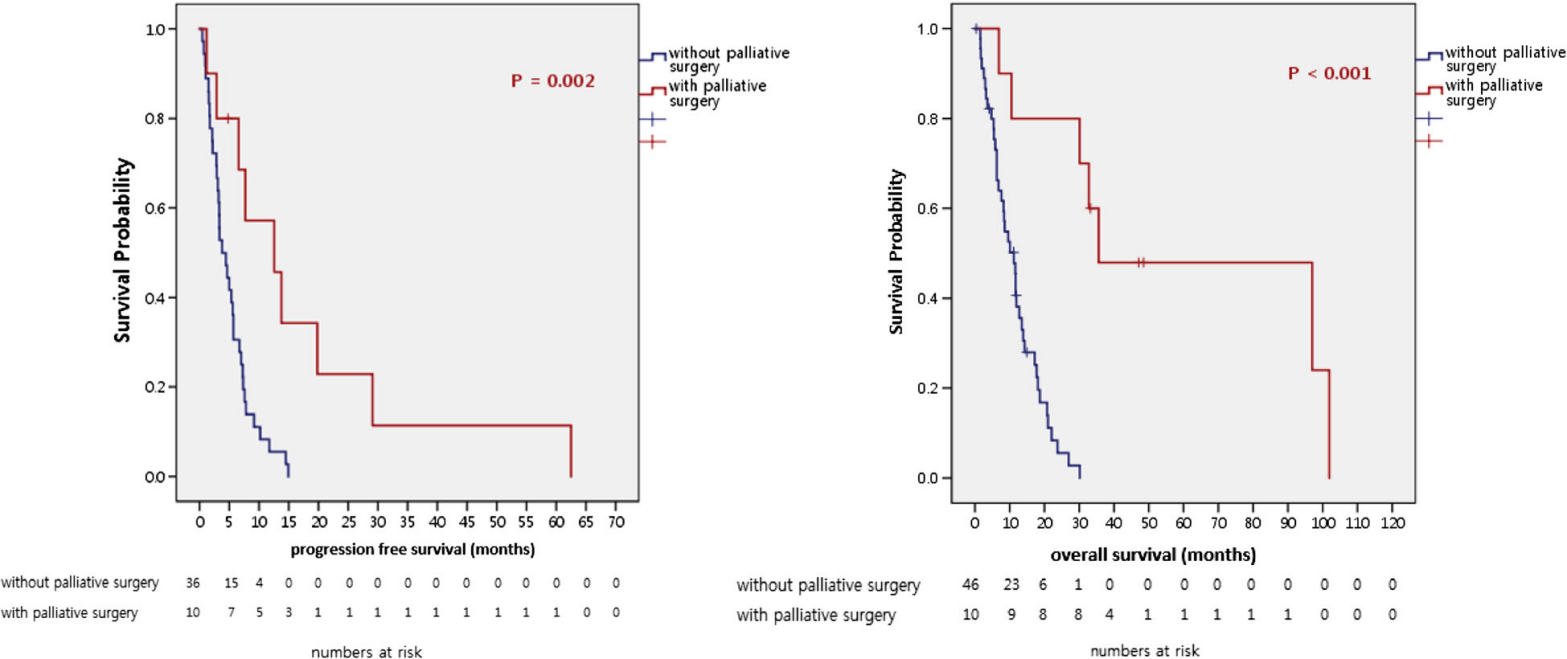
Survival outcomes of 10 patients who received debulking surgery

**Fig. 2:**
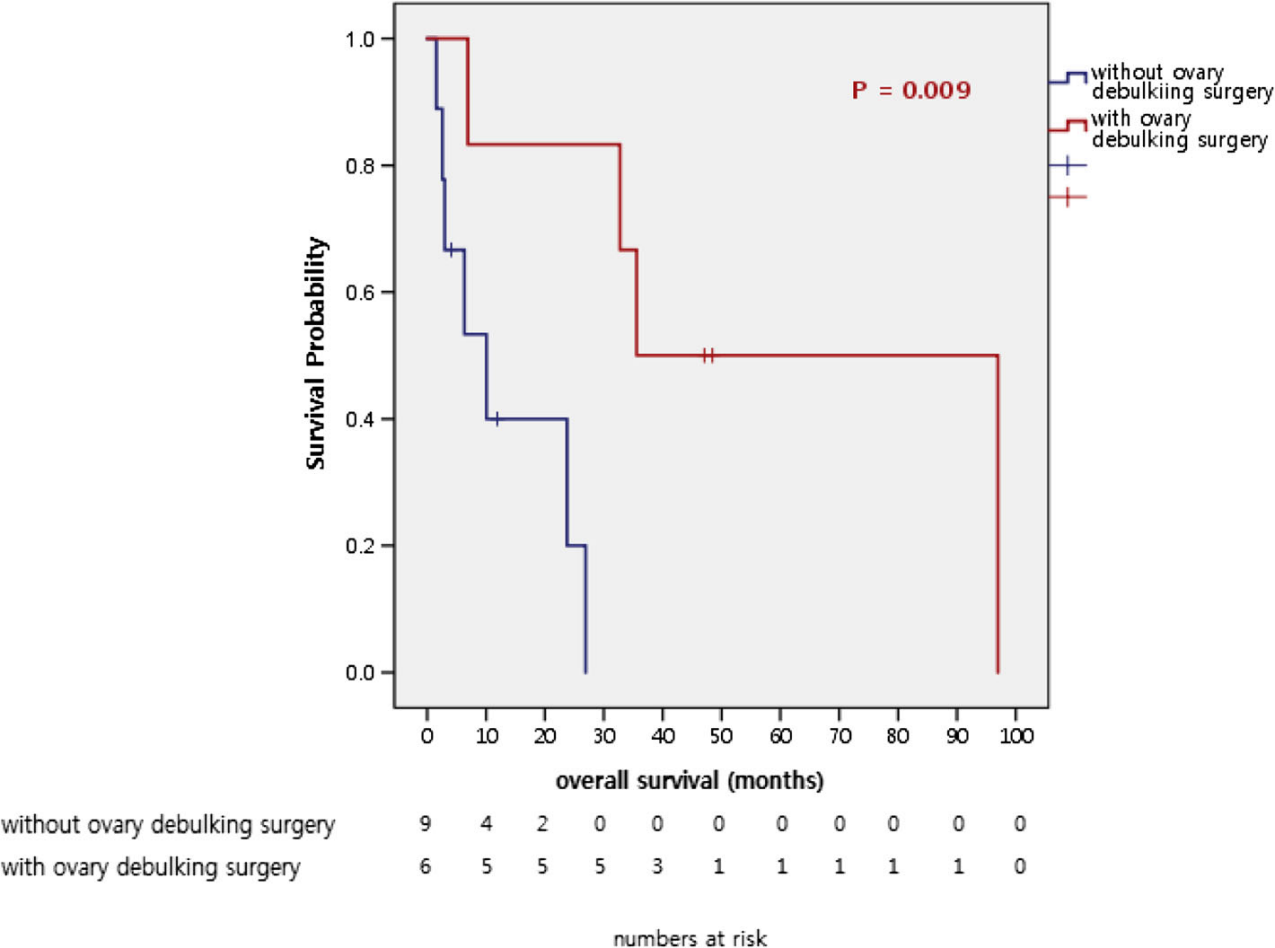
Survival outcomes of 6 patients receiving palliative oophorectomy

Among 56 patients, 1 patient received palliative gastrectomy for palliation of cancer-induced symptoms such as gastric outlet obstruction. Three patients received gastrectomy with curative intent, but they were diagnosed as stage IV GC due to malignant ascites or carcinomatosis peritonei found during surgery. Six female patients received palliative debulking surgery for palliative symptom control for Krukenburg tumor. Ten patients who received palliative surgery showed significantly better first-line PFS (median 3.8 months vs. 12.57 months, respectively; *P* = 0.002) and OS (median 11.2 months vs. 35.57 months, respectively; *P* < 0.001) after recurrence compared to other recurrent or stage IV patients population without palliative surgery (Fig. 1). Comparing six female patients who received palliative surgery to other female patients without surgery, they showed significantly superior OS compared to patients without palliative surgery (median 10.07 months vs. 35.57 months, respectively; *P* = 0.009) (Fig. 2).

## Discussion

The overall incidence of young age GC in Korea was estimated to 3.55 % [13]. In our analysis, the incidence of young age GC was estimated as 3.76 %, similar to national incidence (3.55 %). In overall population, GC is known to have male predominance. However, there was no sexual predominance in our study (Table 1). About half of the patients were diagnosed as SRC, and there were slight female predominance. Previous studies have reported young age GC shows slight female predominance or similar prevalence between both sexes, with higher rate of poorly differentiated carcinoma or SRC by pathologic review [4, 5, 14]. Previously mentioned clinical characteristics were in concordance to studies mentioned above.

The prevalence of early gastric cancer (EGCa) and advanced gastric cancer (AGCa) were similar in our analysis (Table 1). In Western studies, the prevalence of AGCa is by far higher than EGCa [6, 10]. Compared to Western, the prevalence of AGCa and EGCa were similar in Asian studies [4, 5, 14], alike to our analysis. This discrepancy may be due to different screening program between Western and Asia. In Asian countries, active surveillance using upper gastrointestinal endoscopy is performed if patient complains of gastrointestinal symptoms. This active surveillance may have contributed to higher EGCa prevalence compared to Western countries.

Adjuvant chemotherapy was recommended for patients who were diagnosed as stage II or III GC after surgery. S-1 was the most preferred regimen for adjuvant chemotherapy, followed by 5-FU based doublet combination chemotherapy and XELOX regimen. S-1 and platinum doublet regimen was relatively more preferred due to insurance payment issue during follow up period. More advanced stage GC showed higher rate of cancer recurrence, with relatively shorter DFS. 5-year DFS rate in patients who received adjuvant chemotherapy was 50 %. In stage II patients, 5-year DFS rate was estimated to 75 %, and 32 % in stage III patients. Compared to ACTS-GC and CLASSIC study [15, 16], 5-year DFS rates estimated in our analysis showed inferior outcomes. This outcome may be due to heterogeneous adjuvant chemotherapy regimen, but the aggressive clinical behavior of young age GC may have influenced this survival outcome.

The OS of patients with recurrent or metastatic GC was 11.4 months, similar to historical outcomes [17]. Patients with recurrent gastric cancer showed slightly longer median OS-recurrence compared to median OS of metastatic GC, but without statistical significance. Most of the patients received first line to second line palliative chemotherapy.

Among recurrent or metastatic GC patients, patients who received palliative gastrectomy or oophorectomy showed superior survival outcomes compared to recurrent or metastatic GC patients without surgical intervention (Figs. 1 and 2). There are conflicting results about the benefit of gastrectomy and metastasectomy in recurrent or metastatic GC [18]. Prior controversial results may result from heterogeneous patient population, different definitions about palliative surgery and various postoperative systemic treatments. However, recent large studies showed there may be some benefit for survival in patients who received palliative gastrecotmy [19, 20]. Among 10 patients who received palliative surgery in our analysis, 3 patients had metastasis located on peritoneum, without distant metastases. These patients went through subtotal or total gastrectomy with D2 dissection. They showed superior survival outcome compared to patients without surgery, and this result was in concordance to results of Sun et al [19]. One patient received palliative gastrectomy with paraaortic lymph node dissection for symptomatic relief for gastric outlet syndrome and bleeding. After surgery, systemic chemotherapy was administered. He survived for 8 years after initial diagnosis.

Six female patients received palliative oophorectomy for symptomatic control of ovary metastases. They also showed superior survival outcome compared to other female patients with recurrent or metastatic GC with ovary metastases (Fig. 2). The role of palliative oophorectomy in recurrent or metastatic GC with ovary metastases is controversial nowadays, but there are some reports supporting the positive benefit of surgery [21, 22]. The survival gain identified in our analysis is in concordance to prior studies. Among 6 patients in our analysis, 4 patients showed metastases confined to ovary, and palliative systemic chemotherapy was administered after surgery.

The survival gain of palliative surgery in our analysis was in concordance to prior studies discussing the positive role of palliative surgery or metastasectomy in recurrent or metastatic GC [19, 21]. In prior analyses, patient populations were comprised irrespective of age. The positive benefit of palliative surgery or metastasectomy in our study supports the role of surgery in selected patients in young age population. However, considering the number of patients who underwent palliative surgery is small, the role of palliative surgery should be applied with caution in each patient. Patient factors such as performance status, the aim of surgery, response to prior chemotherapy, planned postoperative chemotherapy should be considered when planning palliative surgery. Alike to older GC patient population, metastasectomy might be a treatment option in very carefully selected young age GC patients.

There are some limitations in our analysis. This study was conducted as retrospective manner, and the results of the study should be interpreted in caution. The patient population was selected in single tertiary center, with relatively small numbers of patients observed for survival analysis. This may be connected to selection bias during analysis. However, the incidence of young age GC in our center was similar to nationwide incidence of young age GC. Furthermore, considering most GC patients were referred from local clinic to major tertiary medical centers, the patient population between major medical centers in Korea may be relatively homogeneous in nature. Although the analysis was performed at single tertiary center, the patient characteristics in our analysis may reflect the major characteristics of young age GC in Korea. Based on our analysis, the clinical characteristics of young age GC should be analyzed in multicenter cohort.

## Conclusion

Young age GC was commonly diagnosed at their thirties, without male to female predominance. The prevalence of EGCa and AGCa was relatively similar, and poorly differentiated carcinoma was commonly found compared to the general GC population. The survival outcome of recurrent or metastatic GC was similar to historical data, but locally advanced GC patients showed inferior survival outcomes compared to historical data. Furthermore, in selected patients, palliative surgery may have positive role in survival outcome. A prospective, multicenter study is warranted for further analysis of the clinical characteristics of young age GC in Korea.

## Abbreviations

AGCa: Advanced gastric cancer
EGCa: Early gastric cancer
GC: Gastric cancer
DFS: Disease free survival
OS: Overall survival
PFS: Progression free survival
RECIST: Response evaluation criteria in solid tumors
SRC: Signet-ring cell carcinoma
